# Hematological Normality, Serum Biochemistry, and Acute Phase Proteins in Healthy Beef Calves in the Brazilian Savannah

**DOI:** 10.3390/ani13152398

**Published:** 2023-07-25

**Authors:** Guilherme Augusto Motta, Paulo Sabino Milhomen Neto, Ricardo Perecin Nociti, Áureo Evangelista Santana

**Affiliations:** 1 Department of Veterinary Clinic and Surgery, School of Agricultural and Veterinary Sciences, São Paulo State University, Jaboticabal 14884-900, São Paulo, Brazil; aureo.e.santana@unesp.br; 2 Department of Veterinary Medicine, Araraquara University—UNIARA, Araraquara 14801-340, São Paulo, Brazil; 3 Department of Veterinary Medicine, Federal University of Tocantins, Araguaína 77824-838, Tocantins, Brazil; paulo_sabino_neto@hotmail.com; 4 Department of Biomedicine Veterinary, Faculty of Veterinary Medicine, Montreal University/Saint Hyacinthe, Saint-Hyacinthe, QC J2S 2M2, Canada; rnociti@gmail.com; 5 Laboratory of Molecular Morphophysiology and DevelopmentSchool of Veterinary Medicine and Animal Science, Department of Veterinary Medicine, Faculty of Animal Science and Food Engineering, University of São Paulo, Pirassununga 13635-900, São Paulo, Brazil

**Keywords:** weaning calves, beef breeds, blood count, enzymes, metabolites, proteinogram

## Abstract

**Simple Summary:**

Basal values of hematological and serum biochemical parameters are necessary for health and animal production performance evaluation. Therefore, considering the importance of cattle production in the Brazilian savannah area and the lack of regional data, we aimed to know the differences related to age, breed group, the influence of the productive system, and geographic region. We established baseline values for beef calves at weaning age. Jugular blood samples were collected in a single instant from 30 calves from each of the following breeds: Nellore, Senepol, Nellore × Aberdeen Angus cross, Nellore × Senepol cross, and Nellore × Aberdeen Angus × Senepol cross. Hematological and serum biochemical evaluations and a proteinogram were performed according to routine techniques. In conclusion, the available reference intervals for healthy animals can be used routinely without interference from the geographic region for animals produced without nutritional failures since changes are registered in pathological, infectious, metabolic, or nutritional deficiency situations. Furthermore, these results will help to understand the adaptation advantages and the possible physiological processes of those breeds and their crosses in the savannah biome of Brazil.

**Abstract:**

The Brazilian savannah region, characterized by high average temperatures, well-defined rainy and dry seasons, soil with low productive potential, and high pressure for parasitic diseases, is home to the highest percentage of the beef herd, which is the world’s largest commercial beef producer. Therefore, breeds that present rusticity combined with productivity are the focus of research in cattle breeding in the region. Considering their geographic particularities and their effects on the animals’ blood parameters, the objective was to study the behavior of hematological variables, serum biochemistry, and acute phase proteins in beef calves at weaning age. Jugular blood samples were collected in a single day from 30 weaning calves (about eight months old and 200 kg of body weight) from the following breeds: Nellore, Senepol, Nellore × Aberdeen Angus cross, Nellore × Senepol cross, and Nellore × Aberdeen Angus × Senepol cross. Hematological data were obtained using an automatic cell counter, serum biochemical measurements were obtained using commercial kits, and the electrophoretogram was obtained using the SDS-page technique. In general, the results were consistent with data already published in similar situations regarding health status, age, and level of metabolic activity. However, differences observed between groups can be explained by differences observed in other concurrent variables like temperament. The pure zebu breed was more reactive than pure taurine. While crossbreds showed intermediate values, and parasitic infestation, the pure taurine breed with higher parasite infestation, while the zebu breed had lower values, which produced effects in some laboratory tests, and generated differences between breeds. In conclusion, the reference intervals available for healthy animals can be routinely used without interference from the geographic region for animals produced without nutritional failures as long as changes are recorded in pathological, infectious, metabolic, or nutritional deficiency situations. However, it is suggested that a study covering a larger number of herds may demonstrate a greater geographic effect on the studied variables.

## 1. Introduction

Brazil is the largest exporter of beef in the world [1], and its competitiveness is due to the vast territory suitable for exploitation, mostly hot climate, supply of native plant species consumed by cattle, availability of labor, and the fact that the animals’ diet is mainly composed of green fodder, mainly within the “cerrado”, also known as the Brazilian savannah biome, which is valued by the consumer market [2]. The Brazilian savannah, which covers the states of Bahia, Federal district—Brasília, Goiás, Mato Grosso, Mato Grosso do Sul, Minas Gerais, Paraná, Piauí, São Paulo, and Tocantins, has high average temperatures and very well-defined wet and dry seasons. The Tocantins state has average temperatures between 26.1 and 28 °C, with maximums between 33.1 and 34.2 °C, minimums between 20.1 and 22 °C, and average annual precipitation between 1501 and 1832 mm/year (millimeters/year), and relative air humidity between 65.1 and 70% [3].

The state of Tocantins belongs to the northern region of Brazil and is part of the Tocantins–Araguaia Basin. Its main economic activities are livestock, forestry, and grain production, mainly soy, corn, and rice, with considerable potential for expansion since about 50% of the soil is suitable for agriculture [4]. Currently, the state of Tocantins has 15.18 million hectares of rural area, 56% of which are pastures [5], housing 9.74 million cattle, 8.74 of which are suitable for cutting [6], which is the youngest state in the country.

Laboratory diagnosis consists of comparing the patient’s serum hematological and biochemical values with reference ranges obtained from clinically healthy animals [7,8], there being inherent differences in age, sex, and utility for scientific evaluation, well-being [9], and nutritional assessment [10]. Therefore, such determination should be based on narrow age intervals considering their correlation with the prevalence of determining diseases, such as gastroenteritis in calves [11] and adults [12].

The acute phase reaction occurs in response to infection or tissue damage induced by endogenous or exogenous factors—damage-associated molecular patterns (DAMPs) [13]. It is characterized by fever, leucocytosis, changes in vascular permeability, and metabolic alterations, among other immunological responses. Such phenomena are regulated by a set of plasma proteins, the APPs, which are produced and released into the bloodstream by hepatocytes [14,15].

Adaptive responses to environmental temperature affect productive performance, as nutrients that would be useful for body development and milk production will be diverted to thermoregulatory mechanisms, causing a reduction in dry matter intake, consequently lower rates of daily weight gain and greater susceptibility to infectious and parasitic diseases [16]. Calves until the weaning phase are the category with the highest prevalence of infectious diseases [17], and the use of reference values diverse to the local reality can lead to misinterpretation and, consequently, medical error, which justifies the need for a compilation proper for each laboratory [18]. Based on the aforementioned considerations, we postulated a hypothesis suggesting that the performance of animals reared in the Brazilian savannah may be attributed to breeds that have distinct adaptations to the environment. We propose that comprehending these variations could be achieved through an investigation of hematological parameters, serum biochemistry, and acute phase proteins.

Therefore, this study aimed to determine the mean values for the hematimetric and clinical biochemical variables in calves at the weaning age of the main beef breeds raised in the savannah biome in Brazil.

## 2. Objectives

The primary objective was to determine the baseline values for hematological, biochemical, and metabolic variables and the expression of acute phase proteins (APPs) in healthy weaned beef calves of the main races produced in the state of Tocantins, correlate these data with the degree of endoparasite infestation, the level of reaction to external stimuli, and the concordance of the results with those already demonstrated in the literature.

## 3. Materials and Methods

### 3.1. Experimental Groups

The experimental units consisted of healthy cattle between six and eight months old, in the rearing stage, produced in a single rural establishment located in the municipality of Monte Santo do Tocantins, state of Tocantins, Fazenda Chão Mineiro. The property has 1674.7 ha, an altitude of 300 m above sea level, geographical coordinates of 9°59′56″ S and 49°06′40″ W, average annual rainfall of 2038 mm, and an average temperature of 26.8 °C [19].

Animals were kept on extensively cultivated pastures of *Panicum spp.* and *Brachiaria spp.* in groups whose size was determined by the size of the paddock. Water and commercial 80P (8% of phosphorus) mineral supplement ad libitum and grain concentrate (DM (dry matter): 89.68%, TDN (total digestible nutrients): 65.18%, CP (crude protein): 25%, EE (ethereal extract): 2.44%, Ca (calcium percentage): 1.22%, P (phosphorus percentage): 1.09%), about 1.5 kg/animal/day (0.3% BW/day (bodyweight/day)) daily in the morning, receiving immunization against foot-and-mouth disease, following the sanitary calendar in force, clostridia, and respiratory diseases, as well as endectoparasiticide control with macrocyclic lactones and benzimidazole. Each experimental group corresponded to a breed type: NE (Nellore breed) (n = 31), SE (Senepol breed) (n = 30), NE×AN (double-cross Nellore/Aberdeen Angus) (n = 34), NE×SE (double-cross Nellore/Senepol) (n = 34), and Tricross (triple-cross 50% Senepol, 25% Nellore, 25% Aberdeen Angus) (n = 31), and therefore a total of 160 subjects, and each group had at least 30 uncastrated male individuals subjected to a blood draw at a date close to weaning.

The animals’ health was confirmed by individual inspection in the field, considering that the systemic response to stressful stimuli, such as restraint, is individual and may impair the clinical evaluation [20]. On inspection, the presence of traumatic lesions in the integumentary system, postural alterations suggesting pain, increase in joint regions, exacerbated presence of ectoparasites, ocular secretion, nasal secretion, coughing, alteration in respiratory pattern, claudication, level of consciousness, and abdominal profile (distention or retraction) were verified. At the time of collection of material for blood tests, skin turgor and the presence of enophthalmia were also observed to confirm the state of normal hydration and ocular mucosa coloration. All clinical inspections were performed by an experienced veterinarian. Only those that presented all the before-mentioned parameters within the clinically normal levels for the species [21] were considered healthy and composed the experimental groups. All conduction work, separation, containment, collection of biological material, and veterinary evaluation were conducted within the precepts of ethics and animal welfare with a view to the safety of the animal team.

### 3.2. Behavioral Assessment

Taking into account the diversity of the breed groups that were involved in the study and the possibility of correlation with the hematological and serum biochemical data, a behavioral evaluation was performed to establish a temperament score, from which the methodology was adapted [22]. The adaptation of the method consisted of not measuring the entry and exit speed of the retaining chute using a kinematic evaluation, and the entire evaluation was carried out by the same person at all times. The temperament score was obtained by summing the score applied to the RTS (restraint score) and the ReS (release score). The RTS is categorized in Table 1.

As for ReS, it was measured at the moment of the animal’s release from the cattle crush and consisted of grades 1, 2, 3, 4, and 5 to curb the concentration of intermediate grades. This score is stratified in Table 2.

### 3.3. Sample Collection

Peripheral blood samples were obtained by jugular venipuncture before de-feeding using the BD Vacutainer vacuum system (BD Diagnostics, São Paulo, Brazil) and 40 × 0.9 mm needles preceded by local antisepsis by the same vet every time. Blood aliquots were collected in tubes containing dipotassium ethylenediaminetetraacetic acid (K2EDTA, 7.2 mg), 4 mL, for blood count, and in a 10 mL tube with clot activator for obtaining serum, its biochemical analysis, and protein fractionation [23]. Immediately after collection, the samples were homogenized and packed in a thermal box with reusable ice for transport to the clinical analysis laboratory, processing within a maximum period of 12 h and adequate storage between 2 and 8 °C [24]. To obtain the serum required for biochemical and protein fractionation tests, samples collected in tubes with clot activator were centrifuged at 1800× *g* for 10 min [25] and properly identified in sterile plastic Eppendorf microtubes.

Knowing the influence of gastroenteric parasite load on haematological parameters, serum biochemistry, and inflammatory responses, the animals were submitted to coproparasitological evaluation using stool samples collected directly from the rectal ampulla of each experimental unit, respecting a minimum of 90 days after the last deworming, because this is when macrocyclic lactones and benzimidazoles, in synergistic use, lose their endectocide effect [26]. The samples were identified and stored under refrigeration until the analysis was performed, within a maximum of 12 h.

### 3.4. Laboratory Analysis

#### 3.4.1. Hematology

The blood count included the erythrocyte variables: erythrocyte count (RBC, ×10^6^/µL), free hemoglobin concentration (Hb, g/dL), hematocrit (Ht, %), mean corpuscular volume (MCV, fL), and (MCHC, g/dL); leukocyte variables: total leukocyte count (total Le, ×10^3^/µL), relative (RLF, %), and absolute leucocyte formulae (ALF, ×10^3^/µL); and platelet variables (total platelet count, TPC, ×10^3^/µL), all obtained using an ABX VET automated cell counter ABX VET Horiba (Horiba Abx, Montpelier, France).

The differential leukocyte count (RLF, %) was manual by analysis of blood smear, stained with a mixture of acid and basic anilines, dissolved in methanol [27], and read under light microscopy in immersion objective (100×). The ALF was established by a rule of three using the overall white blood cell (WBC) count and the RLF.

#### 3.4.2. Metabology

The metabolic parameters analyzed, the material, and the methodology used are described in Appendix A, Table A1.

Indirect bilirubin results (mg/dL) were obtained by subtracting the direct bilirubin value from the total bilirubin value. Sodium, potassium, and inorganic calcium values, expressed in mmol/L, were obtained by Roche 9180 Electrolyte Analyser (Roche, São Paulo, Brazil), while the other variables had their results measured with a semi-automatic spectrophotometer LabQuest model (LabTest Diagnóstica S. A., Lagoa Santa, Brazil), and with the respective commercial kits of the same brand.

#### 3.4.3. Proteinogram

The following serum protein fractions were quantified: total protein, ceruloplasmin, haptoglobin, albumin, α1GA, and transferrin, by sodium dodecyl sulfate acrylamide gel electrophoresis (SDS-PAGE) [28]. Following fractionation, the gel was stained with Coomassie blue and plated in 7% acetic acid to remove excess dye until band clarity was achieved. The concentration of each fraction was gauged in a computerized densimeter, as a reference, a marker solution of molecular weights 36, 45, 66, 97.4, 116, and 205 kilodaltons (kDa), as well as purified haptoglobin and α1-antitrypsin.

#### 3.4.4. Coproparasitology

The bovine coproparasitological examination was performed according to the McMaster technique [29], with feces collected from the rectal ampulla of the animals.

#### 3.4.5. Statistical Analysis

All statistical analyses were performed with R software (R Foundation for Statistical Computing, Vienna, Austria). After confirming the homoscedasticity of data by the Levene test and the normality of data by the Cramér Von Mises test, analysis of variance (ANOVA) of quantitative data was done to evaluate the responses. When necessary, a comparison of means of pairs was made using Tukey’s test, with a *p*-value < 0.05. When the data did not meet the requirements of ANOVA, even after data transformation, the non-parametric methodology was used, including the Kruskal–Wallis test with a *p*-value adjusted by the Benjamini–Hochberg method at 5%. The analysis of possible relationships between variables was done with Pearson’s correlation test. The multivariate structure of the data contained in the dataset was explored by dimension reduction (Principal Component Analysis) using the function prcomp from R package stats [30,31,32], grouping, clustering, and data discrimination techniques visualized using by pheatmap R package [33]. Additionally, it was used as the method for combining partial correlation, and an information theory [33] implemented in CeTF Rpackage [34,35].

## 4. Results

The total red blood cell (RBC) count ranged between 10 × 10^6^/µL in the Nellore (NE) group and 8.72 × 10^6^/µL in the Senepol (SE) group (*p* = 1.269 × 10^−4^). The hemoglobin (Hb) concentration was between 11.34 g/dL in the Nellore × Aberdeen Angus cross (NE×AN) group and 10.52 g/dL in the SE group (*p* = 0.0398). The corpuscular hemoglobin concentration (CHC) reached values between 10.68 g/dL in the NE group and 12.37 g/dL in the triple-cross Nellore × Aberdeen Angus × Senepol (Tricross, *p* = 4.3835 × 10^−9^). For mean CHC (MCHC), the values ranged from 33.51% in the NE×AN group to 34.43% in the SE group; there was no difference between the NE, NE×AN, and Tricross groups, but we found a difference between SE and Nellore × Senepol cross (NE×SE).

There was no statistical difference between the groups for hematocrit (Ht) values, which ranged between 33.85 and 30.58%; mean corpuscular volume (MCV) ranged between 31.87 and 36.68 fL, and total platelet count (TPC) ranged between 263.81 × 103/µL and 330.81 × 103/µL. Table 3. brings the average results of the erythrometric variables of the groups.

The restraint score (RTS) ranged from 2.07 in the SE group to 3.39 in the NE group (*p* = 3.33 × 10^−4^), with a significant difference between these and records of different intermediate values in the NE×AN, NE×SE, and Tricross groups. For the release score (ReS), the values ranged from 1.4 (SE group) to 2.39 (NE group) (*p* = 1.705 × 10^−4^), with no difference between the SE, NE×AN, NE×SE, and Tricross groups. Then, the sum of these indexes maintained the same behavior, varying between 3.47 in the SE group and 5.77 in the NE group (*p* = 1.68 × 10^−5^), showing a significant difference, while the other groups presented intermediate values different from the extremes. All the average values by temperament scores are available in Table 4.

Age ranged from 183 (SE) to 242 days (NE) (*p* < 0.05); there was no difference between the NE, NE×AN, NE×SE, and Tricross groups. Birth weight (BrW) showed values between 28.26 (NE×AN) and 31.46 kg (SE group) (*p* < 0.05), whereas the NE, SE, NE×SE, and Tricross groups did not differ. Body weight (BdW) ranged between 181.68 (SE group) and 225.84 kg (Tricross) (*p* < 0.05), while NE and NE×AN, NE×SE, and Tricross did not differ. Mean daily weight gain (MDWG), on the other hand, showed values between 0.71 (NE group) and 0.85 kg/day (Tricross) (*p* < 0.05), and the NE and NE×AN, SE, NE×SE, and Tricross groups showed no significant difference between them, and the medium values are exposed in Table 5.

The counts of parasite eggs and oocysts in the feces showed a little discrepancy between the groups and are described in Table 6. Cestode egg mean counts ranged between 0 epg (eggs per gram of feces, on NE×SE) and 86.66 epg (SE group) feces (epg), and coccidia oocyst mean counts between 20.59 epg (NE×SE) and 51.47 epg (NE×AN), with no statistical difference between the groups. While the trichostrongylid egg means count showed values between 77.94 epg (NE×AN) and 433.33 epg (SE) (*p* = 1.676 × 10^−5^), with only the SE group showing statistical difference compared to the others. Total egg count showed the same behavior as trichostrongylid, varying between 52.94 (NE×SE) and 556.66 epg (SE, *p* = 2.035 × 10^−4^).

Results for leucometric variables are in Table 7, and, total leukocyte (TL) count ranged from 15.23 × 10^3^/µL, NE×AN, to 19.93 × 10^3^/µL, SE (*p* = 8.7694 × 10^−6^), and there was no statistical difference between NE, SE, and Tricross, nor between NE×AN and NE×SE. The total neutrophil count (Neu) ranged between 3.3 × 10^3^/µL in the NE×AN group and 5.89 × 10^3^/µL in the SE group (*p* = 5.7044 × 10^−5^), while the pairs SE and NE×SE, and NE and Tricross did not differ. Total lymphocyte (Lymph) count showed values between 10.41 × 10^3^/µL in the NE×SE group and 13.13 × 10^3^/µL in the Tricross group (*p* = 1.7263 × 10^−5^); there was no significant difference between the NE, SE, and Tricross groups, and between NE×AN and NE×SE. The values observed for total eosinophil count (Eos) ranged between 0.041 × 10^3^/µL, NE×AN and 0.252 × 10^3^/µL, SE (*p* = 0.291), without statistical difference between the NE×AN, NE×SE, and Tricross groups, while the NE and SE groups differed from each other. The total basophil (Bas) count showed results between 0.011 × 10^3^/µL, SE and 0.037 × 10^3^/µL, NE×SE (*p* = 2.2616 × 10^−6^), with only the NE×SE group differing from the others. Total monocyte (Mon) count, on the other hand, showed mean values between 0.54 × 10^3^/µL, NE and NE×SE groups, and 1.01 × 10^3^/µL, SE group (*p* = 4.6739 × 10^−8^), and the NE, NE×AN, and NE×SE groups did not differ from each other, and Tricross and SE differed significantly from all others groups.

Serum activity of the enzymes alanine aminotransferase (ALT), creatinine kinase (CK), and γ-glutamyl transferase (GGT) did not differ significantly between groups, showing values between 17.83 UI/L (SE) and 29.61 UI/L for NE, 244.27 UI/L (SE), 641.55 UI/L (NE×SE), 17.35 UI/L (NE), and 20.80 UI/L for SE. Mean aspartate aminotransferase (AST) results were between 54.32 UI/L (NE×AN) and 69.45 UI/L (Tricross) (*p* = 1.5077 × 10^−4^), with the NE, SE, NE×SE, and Tricross groups not differing from each other. For alkaline phosphatase (AP), values ranged between 431.43 UI/L (SE) and 926.58 UI/L (NE) (*p* = 1.866 × 10^−12^), while SE, NE×AN, NE×SE, and Tricross groups showed no statistically significant difference. The average values of enzyme concentration are in Table 8.

Serum creatinine concentration showed mean values from 1.24 mg/dL in NE×AN to 1.53 mg/dL in NE and Tricross groups (*p* = 1.56358 × 10^−10^), while SE and NE×AN did not differ and NE×SE differed from all others showing intermediate value. For urea concentration, there was variation between 15.94 mg/dL (NE×AN) and 21.73 mg/dL (SE) (*p* = 6.3264 × 10^−5^), while NE, NE×AN, and Tricross did not differ, neither did SE and NE×SE. Cholesterol showed values between 83.87 mg/dL (SE) and 164.32 mg/dL (NE) (*p* < 0.05), and NE, SE, and Tricross differed from all other groups, but NE×AN and NE×SE did not differ. For triglycerides, mean values were observed between 15.80 mg/dL (NE×SE) and 28.55 mg/dL (NE) (*p* = 2.7197 × 10^−9^), and SE, NE×AN, and Tricross groups did not differ. Serum glucose concentration ranged from 91.40 mg/dL, Tricross to 161.03 mg/dL, NE (*p* = 1.1544 × 10^−11^), with no significant difference between SE, NE×AN, and Tricross groups, while the NE×SE group differed from all the others. The total bilirubin value for the groups was between 0.21 mg/dL (NE) and 0.32 mg/dL (SE) (*p* = 1.65 × 10^−3^), the NE×AN, NE×SE, and Tricross groups did not differ from each other or the purebred NE and SE groups. Direct bilirubin varied between 0.08 mg/dL (NE) and 0.26 mg/dL (SE) (*p* = 0); the NE×AN, NE×SE, and Tricross groups did not differ from each other or the purebred groups. Indirect bilirubin showed values between 0.06 mg/dL in SE and NE×SE and 0.13 mg/dL in NE (*p* = 5.5914 × 10^−7^), while NE and Tricross did not differ, nor did the others. Average values are described in Appendix A Table A2.

In general, serum electrolyte concentrations had little variation between groups, and the average values are in Appendix A Table A3. Calcium concentration was between 9.76 mg/dL (NE×SE) and 9.95 mg/dL (NE) (*p* = 0.319), with no significant difference between groups. The ionized calcium concentration showed values between 4.38 mmol/L in Tricross and 4.60 mmol/L in SE (*p* = 0.059), with no significant difference between the groups. Serum chloride was between 99.25 mmol/L (SE) and 160.08 mmol/L (NE×SE) (*p* = 0.229), with no significant difference between groups. Serum iron showed values between 111.71 µg/dL (NE) 160.08 µg/dL (NE×SE) (*p* = 7.9115 × 10^−7^), while NE and Tricross, and SE and NE×SE groups did not differ, and NE×AN differed from all with intermediate value. Serum potassium concentration ranged between 3.78 mmol/L (NE×AN) and 4.15 mmol/L (SE) (*p* = 0.0135), with only NE×AN differing significantly from the others. For serum magnesium, there were mean values between 1.97 mg/dL (SE) and 2.80 mg/dL (NE) (*p* = 0), while NE×SE and Tricross did not differ, and NE×AN differed from the others with intermediate values. For serum sodium concentration, values were determined between 138.47 mmol/L (NE×AN) and 142.40 mmol/L (NE) (*p* = 3.207 × 10^−8^); the NE and NE×SE groups did not differ, as well as SE, NE×AN, and Tricross. For serum phosphorus concentration, the established values were 6.03 mg/dL (NE×AN) and 7.06 mg/dL (NE and Tricross) (*p* = 9.3762 × 10^−9^), while SE, NE×AN, and NE×SE groups did not differ.

Total serum protein concentration showed values between 5.70 g/dL (NE×SE) and 6.41 g/dL (Tricross) (*p* = 9.7105 × 10^−9^), while NE, SE, and NE×AN did not differ with intermediate values. For serum albumin, the mean values varied between 2.92 g/dL (SE) and 3.38 g/dL (NE) (*p* = 0); NE and Tricross did not differ, nor did NE×AN and NE×SE. The mean values obtained for ceruloplasmin concentration are between 9.3 mg/dL (NE×SE) and 13.4 mg/dL (SE) (*p* = 0.013), while the NE, NE×AN, and Tricross groups did not differ from each other, without differing from the extremes. Transferrin demonstrated mean values between 72.5 mg/dL (Tricross) and 129.2 mg/dL (SE) (*p* = 2.6434 × 10^−13^); the Tricross and NE×AN groups did not differ, nor did NE×SE and NE. For haptoglobin, the observed values ranged between 22.1 mg/dL (NE×AN) and 30.6 mg/dL (Tricross) (*p* = 1.466 × 10^−5^), and the NE and Tricross groups did not differ, nor did SE and NE×SE. As for α-1-acid glycoprotein (α1GA), the values were 14.3 mg/dL (NE×AN) and 24.2 mg/dL (Tricross) (*p* = 3.882 × 10^−5^), with SE and Tricross not differing from each other, as well as NE and NE×SE; however, the latter also did not differ from the extremes. Table 9 describes the medium values of the serum proteins of the groups.

**Figure 1:**
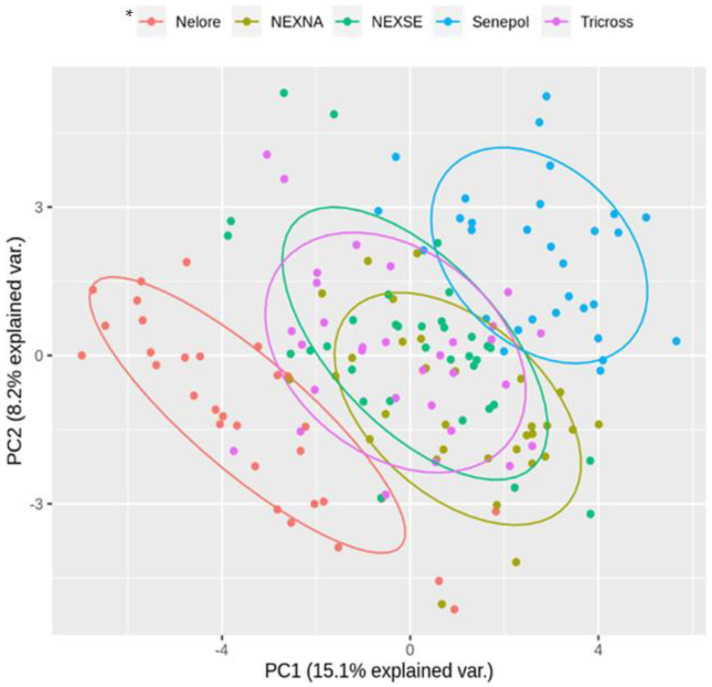
Result of the multivariate statistical analysis represented with principal component analysis (PCA) using the values for hematological and serum biochemical variables of weaning beef calves at Brazilian savannah. * Nelore: Nellore breed, NE×AN: bicrossed by Nellore and Aberdeen Angus, NE×SE: bicrossed by Nellore and Senepol, SE: Senepol breed, Tricross: triple-cross 50% Senepol, 25% Nellore, 25% Aberdeen Angus.

**Figure 2:**
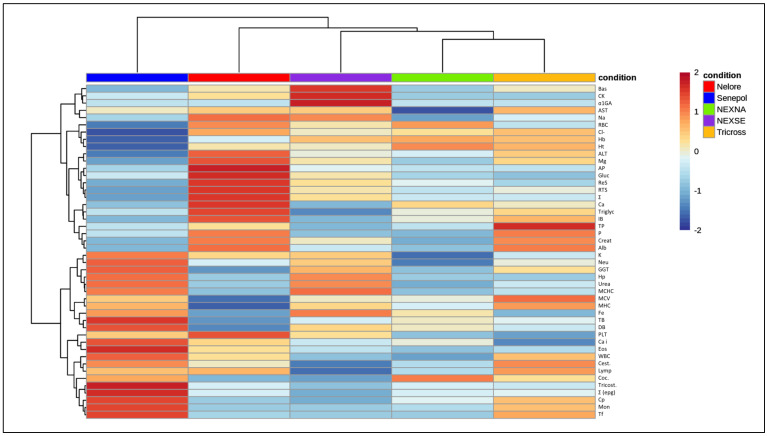
Clustering heatmap by Ward method using mean values of hematological and serum biochemical metabolites from weaning beef calves raised in the Brazilian savannah.

The principal component analysis (PCA), Figure 1, showed a greater global distance between the NE and SE groups, and the crossbreed groups, NE×AN, NE×SE, and Tricross, were close to each other and intermediate to the purebreds, NE and SE. Furthermore, Figure 2 shows the heatmap, in which it is possible to see (columns clusterization by Ward method) the tendency in which SE and NE breeds are the most distant group followed by NE cross breeds. 

Finally, the RIF analysis was significant (absolute rif values > 2) for Fe, Creat, Cpl, and PLT, which can be considered as key metabolites for impacting global difference between the NE group and the SE group, while Tricost and Σ (epg) are responsible for the global difference between SE and NE.

## 5. Discussion

The RBC value showed significant differences only between the Nellore and Senepol breed groups, the crossbreds being similar to pure breeds. Ref. [36] observed in purebred N’Dama animals—a breed that composes the Senepol breed—and its crossbred, lower erythrocyte counts, but pure Brown Swiss animals in the same geographical region and season of the year showed even lower average values. Furthermore, behavior such as phenotype was credited to the Brown Swiss’ greater susceptibility to trypanosomiasis. In general, the RBC value of the Zebu breed does not differ from taurines when compared [36,37,38] or individually [9,39,40,41]; however, it may be suggested that the difference observed in the Nellore breed group may be credited to its high reactivity, a consequently higher stress level, which leads to the splenic and hepatic contractions that occur, directing a greater number of RBCs into the bloodstream [42]. The crossbred animals showed intermediate values to zebuine and taurine for RBC and temperament variables, which suggests the influence of the breed group and intermediate level of the stress response.

According to [16,43], RBC and Ht decrease with heat stress; therefore, there was no such effect in the evaluated breed groups. Except for SE, which presented mean results lower than the others, however, this cannot be considered only as a temperature effect.

The NE group proved to be more responsive to all stimuli considered, corroborating the results of [44,45]. Taurine breeds have lower levels of reactivity to stimuli as well as their crossbreeds [46,47]. Therefore, it can be assumed that the intermediate average values observed in the crossbred groups are due to the heritability of these characteristics, which is significant in the Nellore breed [22]. According to [47], taurine animals with lower degrees of reactivity present better mean daily weight gain (MDWG) and better meat quality, which cooperates with the results obtained. The SE and Tricross groups had the lowest values in temperament evaluations and the best averages for MDWG.

Ht, MCV, and TPC values showed no difference between groups, and their results are similar to those obtained by [37] in Nellore and Holstein breeds, [48] in Norwegian Red, [49] in Pantaneira, and [9] in Hanwoo; while the variables Hb and CHC showed values compatible with similar studies in other races and different age groups [50,51,52]. Age did not influence the behavior of the erythrometric variables between the groups, corroborating the results of [40,49,53,54].

The higher BrW values for the purebred, NE, and SE groups are credited with the fact that these animals are the product of in vitro fertilization since the oocytes that produce such fetuses are exposed to cell growth factors originating from the maturation and in vitro fertilization media, inducing greater fetal growth and eventually overgrowth syndrome [55].

The crossbred groups of Senepol, NE×SE, and Tricross showed higher ADWG than the other groups. Such a difference can be credited to the hybrid vigor by crossing zebuine and taurine breeds, corroborating with [56] and by the thermotolerance inherited from the Senepol breed [57,58]. Moreover, the better average result of the Tricross group is due to, according to [59], better milk production of their double-cross bred mothers NE×AN, which also reflects in the higher weaning weight (BdW) adding value to the product. Therefore, its use is indicated to produce crossbred animals for beef or as embryo recipients. In contrast, the NE×AN crossbreds showed lower daily weight gain, indistinguishable from the NE group. This result is due to the lower tolerance to heat inherited from the Aberdeen Angus breed, characterized by dense black fur [60].

The higher MDWG of SE animals is consistent with the descriptions of [61], who describe their greater thermotolerance, inherited from the African N’Dama, and feed efficiency, inherited from the British Red Poll. The lower result of the NE group compared to the other groups is similar to the results described in the literature for the breed [62,63,64].

Regarding the evaluation of gastrointestinal parasite infestation, no differences were observed in the levels of infestation between the groups NE, NE×AN, NE×SE, and Tricross by the different families of parasites, which can be credited to the zebu heritage [65,66,67]. The SE group showed a quite high average in trichostrongylid and total egg count, which is due to the greater susceptibility inherent in taurine breeds [65,66,67].

Lymphocytes are the dominant cell type in the bovine species, and their count varies with age [68]. The mean values observed for the leukocyte variables are similar to those obtained by [37] in Nellore and Holstein breeds and by [49,69] in the Pantaneira breed, all in the same age group, but with an increase, because it is known that in this age group there is a predominance of lymphocytes [37,49]. Such variation can be credited to the high environmental temperature, which corroborates with [70], who observed that there is higher leukocyte activation in Holstein neonates at different environmental temperatures. However, the similarity between the results of some variables, such as WBC, Neu, and Lymph, between the NE and Tricross groups may be due to the Zebu heritage or an occasional finding, as other variables show similar behavior between the crossbreed and pure breed groups. 

According to [71,72], it can be suggested that SE and Tricross groups had higher TL, Neu, Lymph, and Mon values by their pure taurine and majority taurine breed composition, respectively, with higher susceptibility to infectious diseases; while the double-cross NE×SE and NE×AN show values intermediate to zebuine, NE, and the aforementioned breeds. The high Eos count of the SE group may be associated with higher gastrointestinal parasite infestation, knowing that eosinophils are the cells mainly responsible for the immune response against helminths [73].

Basophils, in general, are responsible for modulating hypersensitivity reactions, inflammatory and autoimmune disorders, and cancer [74]. Therefore, it can be assumed that double-cross NE×SE are more susceptible to allergic disorders; by having a higher Bas count, they can manifest systemic responses of greater intensity.

The activity of serum enzymes ALT, CK, and GGT did not differ between the experimental groups. There were, however, only two points of divergence between the groups. Firstly, the NE×AN group showed lower AST dosage compared to the other groups. According to [75], the elevation of serum AST levels occurs in humans due to physical exertion, muscle injury, infectious diseases, and hepatic lipidosis, for example, which contradicts the obtained result. As there was no concomitant change in the results for the other enzymes, this can be considered an incidental finding. In the NE group, serum AF concentration was twice as high. It is known that this enzyme has an increase in its serum activity associated with muscle injury, physical effort, and stress [76]. This, together with the higher temperament scores concerning the other groups, leads to the conclusion that this increase is due to the muscular effort made during restraint for blood sampling. When compared with published data, the mean values obtained for serum enzyme activity are possibly coincidental since they are different metabolic situations. There was no difference for AST in Curraleiro [77], Girolando [78], and Nellore [79] breeds of the same age and in adult Wagyu cows [80]. However, Ref. [81] found decreased serum AST concentration in Brahman newborn calves, and [81] pointed out elevation due to lactation and gluconeogenesis in Sindi and Girolando cows.

Secondly, as for serum ALT activity, Refs. [82,83] pointed out similar values in Nellore calves of the same age. Regarding GGT, close mean values were described by [77,78,80,84], although the level of metabolic activity greatly influences the mean GGT values leading to an increased in its plasmatic concentration [79,82,85]. Serum AP and CK activity are directly related to muscle activity and phase of bone and muscle growth, respectively [76], which can be observed in the results presented by [85]. In general, the mean values for AP were higher than those observed in the literature when compared to [77,83] in animals of the same age group, while the CK value was similar to that described by [77].

According to [86], there was an increase in AP (10%) and CK (124%) concentrations in cattle transported for about 14 h, and muscle injury is considered an inducer. However, dehydration can cause CK elevation [87]. The animals used in this experiment were submitted to a lower term of water restriction and contention, about five hours, which may have generated the smallest variations observed.

The set of metabolic variables studied showed agreement with the results for the species in different age groups obtained [77,80,81,82,88,88]. The metabolic variables that showed greater differences between the groups were: urea, bilirubin, cholesterol, glucose, and triglycerides. Serum urea and bilirubin levels are known to be related to increased hepatic activity linked to protein synthesis [89]. This increase was observed in the SE group along with higher ADWG, which suggests a positive correlation between the variables.

The NE group presented higher mean values for cholesterol, glucose, and triglycerides, energy metabolites in which serum concentrations are altered by cortisol levels; that is, they increase according to the submission to stressful stimuli [90]. Such increases were observed together with higher temperament score values and greater reactivity to potentially stressful stimuli, which justifies the behavior of these variables compared to the other groups, confirmed by a correlation value equal to 0.51 between glucose and RTS and 0.52 between glucose and ReS.

An inversion between the mean values of indirect and direct bilirubin was only observed in the NE group, which contradicts the results of the other groups and is not described in the literature.

There were a few differences between the groups for the electrolytic variables, which were also observed in the set of metabolic variables and the serum enzymatic activity. These point differences, such as in levels of P, Na, Mg, K, Fe, AST, total protein concentration (TP), creatinine, and albumin, can be diluted with a larger number of samples, whereas works with similar objectives to these have larger sample groups, as in [37], with 360 animals, [91], with 235 animals, and [69], with 300 animals.

The results obtained do not differ from those published by [92] in adult Brahman females, [93] in adult Senepol females and heifers, [93] in adult Holstein crossbred females, and [77] in Curraleiros of the same age. However, there are alterations caused by pathological situations. Ref. [94] observed a reduction in serum P levels in African nomadic herds but with the maintenance of Fe, Mg, and Na levels. Similarly, Ref. [95] recorded a reduction in all serum minerals in non-supplemented herds on the Nigerian savannah. In hypocalcaemic Holstein cattle with ketosis or metritis, Ref. [96] obtained no change in Cl, Na, and K levels, but Ca concentrations always presented below the control group.

According to [97], helminthiasis does not alter serum P levels, and there is a direct effect of supplementation on this variable, which corroborates with [93], when Fe, Mg, and P levels oscillate according to the season, i.e., according to the quality of forage available, justifying the chronic use of supplementation.

The concentration of total serum proteins corresponds to the sum of serum albumin, α-, β-, and γ-immunoglobulins and APPs [98]. Lower values were observed in the taurine, SE, NE×AN, and NE×SE breed composition groups. This is related to the lower serum albumin levels recorded in the same groups, which in turn are reduced because they are categorized as more susceptible to gastrointestinal parasitosis due to spoliation [66,67,68].

The mean values of TP and albumin of the NE and Tricross groups follow the results obtained by [99] in Guzerá cattle, [100] in Brahman, and [77] in Curraleiros, of the same age, while the results of the SE, NE×AN, and NE×SE groups resemble those obtained by [101] in Aberdeen Angus cattle, Hereford and their crossbreeds, [102] in taurine dairy cows, and [103] in taurine beef and dairy cattle.

The concentrations of APPs in the SE, NE×AN, and NE×SE groups proved to be higher compared to the pure zebu, NE, due to their greater inflammatory response to these parasitic agents, corroborating with [104] in lactating Holstein females. Ref. [105] observed that the expression of APPs changes according to the degree of infestation by *Rhipicephalus (Boophilus) microplus* ticks, decreasing appetite and leading to reduced weight gain, which may relate to the lower hardiness of taurine breeds. Parallel to the depression of appetite, these animals mobilize greater amounts of amino acids and energy for the synthesis of pro-inflammatory molecules, which reduces their efficiency within production systems where the challenge by pathogens and parasites is greater [105].

Therefore, it can be concluded that Zebu animals and some of their more rustic crosses tend to have higher levels of TP due to the higher concentration of albumin and lower concentrations of APPs due to their lower susceptibility to diseases and, therefore, greater rusticity, while opposite behaviors are observed in taurine breeds and their crosses less resistant to stress factors.

Ceruloplasmin (Cpl) is a protein produced by hepatocytes carrying copper (40 to 70% of plasma copper) [106]. Its main functions are copper transport, regulation of plasma iron concentration, oxidation of organic amines, ferroxidase activity, and prevention of free radical formation [107]. It is an acute phase reactant in inflammation, infection, trauma, diabetes, and pregnancy with antioxidant properties [108], showing a response pattern characterized as moderate [109].

Among the experimental groups, there was little difference in the values of Cpl concentration. SE showed higher Cpl concentrations, which may relate to the lower hardiness inherent in taurine breeds, accompanied by the NE×AN and Tricross groups. However, the NE×SE group showed a lower value than the others and is statistically similar to the value of the NE group, which suggests that this group has significantly inherited the hardiness of the Nellore and Senepol breeds, which, despite being taurine, is recognized for both. Ref. [110] pointed out stress as the main factor inducing the elevation of the serum Cpl concentration in taurine cattle, but with higher basal values (24 mg/dL) than those observed in the groups studied (between 9.3 and 13.4 mg/dL). In contrast, Ref. [111] found a value of 6 mg/dL in adult Holstein cattle in Iran.

Ref. [112] designate Cpl as an important diagnostic marker for a cupric deficiency when its serum concentration decreases, which should not be considered for these experimental groups since they receive ad libitum mineral supplementation. The reduction in Cpl activity in adequately nourished animals, on the other hand, may be attributed to age, being reduced in young animals [113].

Transferrin (Tf) is an APP responsible for chelating free iron, making it unavailable for microbial metabolism [114]. The increase in its serum concentrations during the inflammatory phase contributes to the process of the non-specific immune response against pathogens, as it impairs the replication process of some viruses, bacteria, and fungi in the tissues [115]. However, Ref. [116] observed no statistical difference between healthy animals and those with respiratory system infections.

Among the groups, the purebreds NE and SE showed higher serum Tf concentrations, which may confer greater resistance to microbial diseases to zebuine breeds and African taurines, such as N’Dama, that contributed to the formation of the Senepol breed. The crossbred groups, NE×AN, NE×SE, and Tricross, showed lower results than the purebred breeds: NE×SE showed higher values than the groups with a breed percentile of Aberdeen Angus, a British taurine breed of known superior susceptibility to microbial diseases. No baseline values for serum Tf concentration were found in the species, but Refs. [114] and [116] pointed out that animals up to one year of age tend to have higher values due to the greater susceptibility of this range to infections.

Haptoglobin (Hp) is a very important major expression pattern APP for ruminant species [117]. Its functions are to bind to free hemoglobin during hemolytic processes, making its iron ion unavailable for microbial metabolism, partially inhibit bilirubin synthesis, and stimulate macrophage pathogen recognition function [118]. According to [119], Hp has diagnostic and prognostic importance in cases of respiratory infection in confined animals, increasing rapidly in acute cases and reducing its activity after antimicrobial treatment. Thus, its expression pattern may signify enhanced immunocompetence and disease resistance in cattle [120], corroborating with [121], who observed that bovine concentrations were minimal in healthy cattle, intermediate in animals submitted to treatment, and high in those with relapses.

However, the results of the experimental groups may not coincide with published descriptions of studies with sick animals. The NE group showed higher baseline values, followed by Tricross, SE, NE×AN, and NE×SE, respectively. Knowing the lower susceptibility to disease of zebuine animals, it can be suggested that they present higher basal values, but their response to infection corresponds to that described by [119,121].

The mean values observed are similar to those obtained by [122] in adult and young Holstein cattle, [123] in young taurine cattle in the finishing phase, and [124] in adult Zebu and Holstein crossbreeds. This suggests that there is no age or breed influence on the baseline value of the serum Hp concentration but rather the influence of the occurrence of diseases of an infectious character [125].

The α1GA, on the other hand, has anti-inflammatory and immunomodulatory actions, mainly inhibitory for neutrophils and the complement system, and maintenance of vascular permeability [126], transport of small hydrophobic molecules [127], being a very important carrier protein binding to more than 300 different molecules [128]. In cattle, it is anti-inflammatory, modulates mononucleotide and neutrophilic activity, reactive oxygen species production, and degranulation [129]. According to [130], α1GA reduces the rate of apoptosis of monocytes, which are responsible for removing leukocytes from the inflammatory focus. Therefore, it can be suggested that animals with higher plasma concentrations during the pathological process have a shorter convalescence period and faster tissue recovery.

The SE, NE×SE, and Tricross groups showed higher serum α1GA concentrations, while NE×AN showed the lowest value and NE intermediate to the others. This behavior may indicate that the Senepol breed has a less intense inflammatory response, which is transmitted to its crossbreeds. This trait may have an opposite behavior about the Aberdeen Angus breed since its double-cross breed with Nellore showed a lower average value. The mean values for serum α1GA concentration of the experimental groups are similar to those observed by [130] in taurines of different ages, those demonstrated by [131,132] in adult Holstein cows, and in healthy and with subclinical mastitis Simental cows [133,134].

The global distance of the mean values between the NE and SE groups shows that a pure breed stands out for its rusticity, NE, and the other, SE, for its productive potential. Considering the extensive production system applied in the Brazilian savannah region and the NE breed available [1], combined with the diffusion of the FTAI technique using semen of taurine breeds, all breed types evaluated in the work have a favorable potential for production since the level of technicality of the activity in the herd will decide which breed type is more suitable, more rustic, or more productive.

The trend between the averages shows that NE×AN and Tricross are closer and, therefore, tend to present closer results. Even though Tricross has superior MDMG than all groups, which is credited to the milk production potential of its NE×AN bi-crossbred hand [58], it is also noted that the means of the NE group tend to approach the means of the crossbred groups, which may be a product of the greater heritability of their characteristics in the process of miscegenation.

NE has lower levels of Cpl; however, it also shows lower serum concentrations of Fe, which may contribute to its greater resistance to infectious processes. Additionally, the concentration of serum Fe may be under the influence of Tf, which also has a regulating function of serum iron levels [114], since the group showed an average value in the second group more distant from all other groups. On the other hand, the SE group has an average value for serum Fe higher than all groups since, as a taurine descendent breed, it tends to be more susceptible to infections. Although Creat and PLT are responsible for differentiating the means of the NE group compared to the others, no pathophysiological justification was found. Therefore, it can be judged that this is an occasional finding.

For the SE group, the mean values of Tricoss. (epg) and Σ (epg) were responsible for distancing from the others. Being a pure taurine breed, such results were not surprising, considering the breed group’s greater susceptibility to parasite infestation [65,66,67].

## 6. Conclusions

Most hematological variables, serum biochemistry, and APPs concentrations were not influenced by breed. However, temperament and parasitic infestation produced effects in some laboratory tests, which generated differences between breeds. There is a consistent literature record on the effect of age, mainly on hematological data. However, the repetition of the analyses with a larger sample universe, staggered at various moments with the same experimental units, may show differences compatible with those demonstrated in the literature considering the alterations in the variables studied due to age, corroborating the literature that deals with older animals. 

Taking together the present results, we suggest that parasite resistance may be a key factor in adaptation to the Brazilian savannah. In conclusion, all breed types evaluated in the work were healthy and had a favorable potential for production. The level of technicality of activity in the herd will decide which breed type is more suitable, more rustic, resistant to parasites, and therefore more productive.

## Figures and Tables

**Table 1:** Categorization of the restraint score (RTS) adapted from [22].

Score	Characteristics
1	Absence of resistance.
2	The animal shows some movement and keeps its head and ears erect.
3	The animal presents frequent and not vigorous movement, with head, tail, and ear movements, in addition to exposure to the sclerotic membrane.
4	The animal offers great resistance, abrupt and vigorous movement of the head, tail, and ears, exposure of the sclera, audible breathing, and the possibility of jumping or falling.

RTS: restraint score.

**Table 2:** Categorization of the release score (ReS) adapted from [22].

Score	Characteristics
1	The animal leaves the cattle crush walking slowly and allowing less than two meters approach to the observer.
2	The animal trots or runs for a few seconds and allows two to three meters approach by the observer.
4	The animal runs intending to escape, moving its tail intensely and not allowing any approach.
5	Animal runs, throwing itself against fences, threatening or attacking the observer.

ReS: release score.

**Table 3:** Maximum, mean, standard deviation, median and minimum values for erythrometric variables and platelet count of weaning beef calves at Brazilian savannah.

		Erythrometric Variables and Platelet Count						
Breed		RBC (×10^6^/µL)	Hb (g/dL)	Ht (%)	MCV (fL)	MCH (g/dL)	MCHC (%)	PLT (×10^3^/µL)
NE	Min	7.52	7.8	25.4	27.5	9.3	308	119
	Mean	10.0 *^,a^	11.01 ^ab^	32.75 ^a^	31.87 ^a^	10.68 ^b^	335.61 ^b^	330.81 ^a^
	SD	2.25	1.38	3.69	2.59	0.74	11.42	114.24
	Median	10.24	11.1	33.4	31.8	10.6	335	326
	Max	13	14.3	42.7	36.8	12.3	356	571
SE	Min	5.74	8.2	24	28.8	10.2	331	74
	Mean	8.72 ^b^	10.52 ^b^	30.58 ^a^	35.50 ^a^	12.23 ^a^	344.27 ^a^	308.23 ^a^
	SD	1.44	1.17	3.49	3.59	1.26	8.02	120.48
	Median	8.83	10.35	29.75	34.5	11.85	344	295
	Max	11.54	12.6	36.6	41.8	14.7	364	513
NE×AN	Min	4.76	8.7	26.7	26.3	9.2	315	24
	Mean	9.75 ^a^	11.34 ^a^	33.85 ^a^	35.55 ^a^	11.87 ^a^	335.15 ^b^	271.94 ^a^
	SD	1.58	1.07	3.12	6.51	1.94	11.13	143.41
	Median	9.74	11.2	33.65	34.35	11.45	333.5	292
	Max	12.93	13.5	41.9	38.5	20.7	356	574
NE×SE	Min	7.16	9	26.5	25.9	9.4	325	87
	Mean	9.44 ^ab^	11.24 ^a^	32.58 ^a^	34.75 ^a^	11.98 ^a^	344.97 ^a^	298.41 ^a^
	SD	1.09	0.97	2.80	3.30	1.09	9.58	108.56
	Median	9.39	11.25	32.6	34.6	12.05	344.5	296.5
	Max	11.76	13.5	39.4	36.1	15.4	362	568
Tricross	Min	7.49	9.4	27.1	31	10.5	317	41
	Mean	9.14 ^ab^	11.26 ^a^	33.35 ^a^	36.68 ^a^	12.37 ^a^	337.48 ^b^	263.81 ^a^
	SD	1.02	0.99	2.91	2.79	0.88	8.90	120.09
	Median	8.92	11.5	34.2	37.1	12.3	338	256
	Max	12.06	13.4	38.7	41.4	14	359	560

* Mean values followed by different letters in the columns differ by the Kruskall–Wallis test with *p* adjusted by the Benjamini–Hochberg method at 5% (*p* ≤ 0.05). Hb: hemoglobin concentration, Ht: hematocrit, Max: maximum value, Median: median value, Min: minimum value, MCHC: mediun corpuscular hemoglobin concentration, MCV: medium corpuscular volume, MCH: medium corpuscular hemoglobin, NE: Nellore breed, NE×AN: bicrossed by Nellore and Aberdeen Angus, NE×SE: bicrossed by Nellore and Senepol, PLT: platelet count, RBC: red blood cell count, SE: Senepol breed, SD: standard deviation, Tricross: triple-cross 50% Senepol, 25% Nellore, 25% Aberdeen Angus.

**Table 4:** Average values ± standard deviation of RTS (restraint score), ReS (release score), and Σ (summation) for temperament avaliation of weaning beef calves at Brazilian savannah.

	Temperament Variables		
Breed	RTS	ReS	Σ
NE	3.39 ± 0.99 *^,a^	2.39 ± 1.02 ^a^	5.77 ± 1.65 ^a^
SE	2.07 ± 0.98 ^c^	1.40 ± 0.50 ^b^	3.47 ± 1.14 ^c^
NE×AN	2.56 ± 1.26 ^bc^	1.70 ± 0.87 ^b^	4.26 ± 1.76 ^bc^
NE×SE	2.70 ± 1.14 ^b^	1.94 ± 1.23 ^ab^	4.64 ± 2.03 ^b^
Tricross	2.61 ± 1.08 ^bc^	1.55 ± 0.81 ^b^	4.16 ± 1.61 ^bc^

* Mean values followed by different letters in the columns differ by the Kruskall–Wallis test with *p* adjusted by the Benjamini–Hochberg method at 5% (*p* ≤ 0.05). NE: Nellore breed, NE×AN: bicrossed by Nellore and Aberdeen Angus, NE×SE: bicrossed by Nellore and Senepol, ReS: release score, RTS: restraint score, SE: Senepol breed, Tricross: triple-cross 50% Senepol, 25% Nellore, 25% Aberdeen Angus, Σ: summation.

**Table 5:** Average values ± standard deviation of age, birth weight, body weight at weaning, and daily medium gain of weaning beef calves at Brazilian savannah.

Breed	Age (Days)	BthW (kg)	BW (kg)	MDMG (kg/day)
NE	242.26 ± 6.87 *^,ab^	31.29 ± 1.75 ^a^	202.22 ± 22.77 ^b^	0.71 ± 0.09 ^b^
SE	183.47 ± 5.18 ^d^	31.46 ± 1.25 ^a^	181.68 ± 20.60 ^c^	0.81 ± 0.11 ^a^
NE×AN	236.26 ± 6.59 ^bc^	28.26 ± 1.58 ^c^	197.73 ± 26.45 ^b^	0.72 ± 0.11 ^b^
NE×SE	243 ± 22.00 ^a^	28.91 ± 1.14 ^bc^	218 ± 29.0 ^a^	0.79 ± 0.16 ^a^
Tricross	231.32 ± 12.39 ^c^	29.26 ± 2.03 ^b^	225.84 ± 30.97 ^a^	0.85 ± 0.15 ^a^

* Mean values followed by different letters in the columns differ by the Kruskall–Wallis test with *p* adjusted by the Benjamini–Hochberg method at 5% (*p* ≤ 0.05). BthW: birth weight, BW: body weight, kg: kilogram, MDMG: mean daily medium gain, NE: Nellore breed, NE×AN: bicrossed by Nellore and Aberdeen Angus, NE×SE: bicrossed by Nellore and Senepol, SE: Senepol breed, Tricross: triple-cross 50% Senepol, 25% Nellore, 25% Aberdeen Angus.

**Table 6:** Average values ± standard deviation of egg count of trichostrongylides, coccidia, cestodes, and summation of weaning beef calves at Brazilian savannah.

	Types of Endoparasites			
Breed	Tricost. (epg)	Cest. (epg)	Coc. (epg)	Σ (epg)
NE	85.48 ± 130.51 *^,b^	35.48 ± 164.41 ^a^	22.58 ± 59.93 ^a^	143.55 ± 208.06 ^b^
SE	433.33 ± 560.68 ^a^	86.66 ± 446.81 ^a^	36.66 ± 64.24 ^a^	556.66 ± 832.19 ^a^
NE×AN	77.94 ± 77.06 ^b^	20.59 ± 120.05 ^a^	51.47 ± 155.45 ^a^	150 ± 219.16 ^b^
NE×SE	32.35 ± 63.82 ^b^	0	20.59 ± 52.39 ^a^	52.94 ± 77.79 ^b^
Tricross	74.19 ± 113.92 ^b^	54.84 ± 199.73 ^a^	29.03 ± 60.24 ^a^	158.06 ± 221.77 ^b^

* Mean values followed by different letters in the columns differ by the Kruskall–Wallis test with *p* adjusted by the Benjamini–Hochberg method at 5% (*p* ≤ 0.05). Cest.: *Cestoda*, Coc.: coccidia, epg: eggs per gram of feces, NE: Nellore breed, NE×AN: bicrossed by Nellore and Aberdeen Angus, NE×SE: bicrossed by Nellore and Senepol, SE: Senepol breed, Tricost.: *Trichostrongylidae*, Tricross: triple-cross 50% Senepol, 25% Nellore, 25% Aberdeen Angus, Σ: summation.

**Table 7:** Maximum, mean, standard deviation, median, and minimum for differential white blood cell count of weaning beef calves at Brazilian savannah.

		Types of Leucocytes					
Breed		WBC (×10^3^/µL)	Neu (×10^3^/µL)	Lymp (×10^3^/µL)	Eos (×10^3^/µL)	Bas (×10^3^/µL)	Mon (×10^3^/µL)
NE	Min	11.81	2.35	7.82	0.01	0	0.07
	Mean	17.90 *^,a,b^	4.28 ^bc^	12.92 ^a^	0.137 ^b^	0.022 ^b^	0.54 ^c^
	SD	3.90	1.20	3.28	0.132	0.013	0.16
	Median	17.97	4.06	12.48	0.1	0.1	0.55
	Max	29.32	7.92	22.8	0.63	0.04	0.83
SE	Min	13.28	1.80	7.25	0.02	0	0.18
	Mean	19.93 ^a^	5.89 ^a^	12.68 ^a,b^	0.252 ^a^	0.011 ^b^	1.01
	SD	5.12	3.30	2.56	0.212	0.011	0.57 ^a^
	Median	19.60	5.30	12.75	0.21	0.01	1.10
	Max	38.91	17.25	19.55	0.84	0.04	2.35
NE×AN	Min	10.91	1.15	7.84	0	0	0.17
	Mean	15.23 ^c^	3.30 ^c^	11.30 ^bc^	0.041 ^c^	0.012 ^b^	0.58
	SD	2.85	1.37	2.30	0.023	0.011	0.19 ^c^
	Median	14.48	2.82	10.82	0.04	0.01	0.55
	Max	21.92	6.46	17.60	0.11	0.05	1.03
NE×SE	Min	9.92	1.56	6.37	0	0	0.05
	Mean	16.03 ^b,c^	4.96 ^a,b^	10.41 ^c^	0.073 ^c^	0.037 ^a^	0.54
	SD	3.69	1.92	2.80	0.07	0.032	0.27 ^c^
	Median	15.38	4.82	9.90	0.05	0.03	0.5
	Max	29.96	10.49	22.28	0.3	0.16	1.21
Tricross	Min	11.04	2.2	5.84	0.01	0	0.33
	Mean	18.57 ^a^	4.48 ^b^	13.13 ^a^	0.068 ^c^	0.021 ^b^	0.86 ^b^
	SD	4.11	1.83	3.23	0.05	0.018	±0.36
	Median	18.18	4.05	12.57	0.05	0.02	0.86
	Max	26.98	10.16	20.95	0.22	0.07	1.89

* Mean values followed by different letters in the columns differ by the Kruskall–Wallis test with *p* adjusted by the Benjamini–Hochberg method at 5% (*p* ≤ 0.05). Bas: basophils count, Eos: eosinophils count, Lymp: lymphocytes count, Max: maximum value, Median: median value, Min: minimum value, Mon: monocytes count, NE: Nellore breed, NE×AN: bicrossed by Nellore and Aberdeen Angus, NE×SE: bicrossed by Nellore and Senepol, Neu: neutrophils count, SE: Senepol breed, SD: standard deviation, Tricross: triple-cross 50% Senepol, 25% Nellore, 25% Aberdeen Angus, WBC: white blood cells count, µL: microliter.

**Table 8:** Maximum, mean, standard deviation, median, and minimum for serum enzyme concentration of weaning beef calves at Brazilian savannah.

		Enzymes				
Breed		ALT (U/L)	AST (U/L)	CK (U/L)	AP (U/L)	GGT (U/L)
NE	Min	18	19	109	450	10
	Mean	29.61 *^,a^	69.22 ^a^	433.84 ^a^	926.58 ^a^	17.35 ^a^
	SD	5.54	21.22	280.68	296.44	3.78
	Median	29	65	323	917	17
	Max	44	126	1187	1735	24
SE	Min	12	28	99	287	14
	Mean	17.83	66.43 ^a^	244.27 ^a^	431.43 ^b^	20.80 ^a^
	SD	4.85 ^a^	21.71	159.1	109.14	5.24
	Median	17	63	197	404.5	20
	Max	40	154	5695	758	40
NE×AN	Min	8	33	99	324	9
	Mean	26.13 ^a^	54.32 ^b^	278.29 ^a^	461.32 ^b^	18.03 ^a^
	SD	37.36	10.96	292.92	97.71	5.74
	Median	19	56	178	444	17
	Max	226	74	1586	781	39
NE×SE	Min	13	45	89	246	13
	Mean	23.94 ^a^	69.41 ^a^	641.55 ^a^	512.44 ^b^	19.85 ^a^
	SD	7.13	28.03	993.49	132.59	4.06
	Median	22.5	66	255	515	19.5
	Max	53	214	2840	790	31
Tricross	Min	16	48	121	257	11
	Mean	25.90 ^a^	69.45 ^a^	294.45 ^a^	459.32 ^b^	19.45 ^a^
	SD	4.57	11.18	331.50	125.80	5.50
	Median	26	68	179	466	18
	Max	37	91	1659	760	36

* Mean values followed by different letters in the columns differ by the Kruskall–Wallis test with *p* adjusted by the Benjamini–Hochberg method at 5% (*p* ≤ 0.05). ALT: alanine amino transferase, AP: alkaline phosphatase, AST: aspartate amino transferase, CK: creatinine kinase, GGT: γ-glutamyl transferase, Max: maximum value, Median: median value, Min: minimum value, NE: Nellore breed, NE×AN: bicrossed by Nellore and Aberdeen Angus, NE×SE: bicrossed by Nellore and Senepol, SD: standard deviation, SE: Senepol breed, Tricross: triple-cross 50% Senepol, 25% Nellore, 25% Aberdeen Angus.

**Table 9:** Maximum, mean, standard deviation, median, and minimum for serum acute phase proteins and total protein concentrations of weaning beef calves at Brazilian savannah.

		Breed				
APP’s		NE	SE	NE×AN	NE×SE	Tricross
TP (g/dL)	Min	5.4	4.8	4.7	4.9	5.8
	Mean	6.02 *^,b^	5.84 ^bc^	5.83 ^bc^	5.70 ^c^	6.41 ^a^
	SD	0.42	0.37	0.37	0.40	0.38
	Median	5.9	5.9	5.8	5.7	6.4
	Max	6.9	6.6	6.6	6.6	7
Alb (g/dL)	Min	3	2.6	2.4	2.6	3
	Mean	3.38 ^a^	2.92 ^c^	2.95 ^b,c^	3.04 ^b^	3.36 ^a^
	SD	0.23	0.16	0.20	0.19	0.19
	Median	3.4	2.9	3	3.1	3.4
	Max	3.9	3.2	3.3	3.5	3.7
Cpl (mg/dL)	Min	2.08	5.4	3.66	3.17	4.16
	Mean	9.9 ^a,b^	13.4 ^a^	10.8 ^a,b^	9.3 ^b^	11.9 ^a,b^
	SD	4	6	3	3	4
	Median	9.57	11.53	11.1	8.6	11.81
	Max	24.27	35.03	19.17	21.65	23.59
Tf (mg/dL)	Min	36.14	99.13	43.47	57.78	34.68
	Mean	118.6 ^b^	129.2 ^a^	79.8 ^c^	96.6 ^b^	72.5 ^c^
	SD	48	22	23	21	21
	Median	113.04	126.75	75.31	93.14	69.23
	Max	242.4	176.12	136.65	144.67	133.08
Hp (mg/dL)	Min	18.97	14.78	10.54	6.72	19.32
	Mean	30.1 ^a^	25.3 ^b^	22.1 ^c^	23.1 ^b,c^	30.6 ^a^
	SD	7	5	5	8	9
	Median	27.97	26.16	22.08	25.09	26.16
	Max	52.05	39	32.62	41	62.71
α1GA (mg/dL)	Min	9.08	7.59	5.42	4.37	9.6
	Mean	17.7 ^b,c^	22.3 ^a^	14.3 ^c^	20 ^a,b^	24.2 ^a^
	SD	6	6	5	11	11
	Median	16.2	22.7	13.66	21.92	20.77
	Max	32.53	36.52	30.99	43.75	54.26

* Mean values followed by different letters in the lines differ by the Kruskall–Wallis test with *p* adjusted by the Benjamini–Hochberg method at 5% (*p* ≤ 0.05). Alb: albumin, APP’s: acute phase proteins, Cpl: ceruloplasmine, g/dL: gram/deciliter, Hp: haptoglobine, Max: maximum value, Median: median value, Min: minimum value, mg/dL: milligram/deciliter, NE: Nellore breed, NE×AN: bicrossed by Nellore and Aberdeen Angus, NE×SE: bicrossed by Nellore and Senepol, SE: Senepol breed, SD: standard deviation, Tf: transferrine, Tricross: triple-cross 50% Senepol, 25% Nellore, 25% Aberdeen Angus, α1GA: acid glycoprotein α1.

## Data Availability

The data presented in this study are available upon request from the corresponding author. The data are not publicly available due to privacy or ethical restrictions, and the data that support the findings of this study are available in this article.

## Appendix A

Some tables referring to the methodology and results are in this Appendix A.

**Table A1:** Metabolic parameters, methodologies, and units used.

Metabolic Variable	Methodology	Unity
Albumin	Bromocresol green method	g/dL
ALT	UV kinetic method	U/L
AST	UV kinetic method	U/L
Bilirubin (total and indirect)	Sims-Horn method	mg/dL
Calcium	O-cresolphthalein method	mg/dL
Inorganic calcium	Electrolyte analyser *	mmol/L
CK	CK	U/L
Chloride	Mercury thiocyanate method	mEq/L
Cholesterol	Enzymatic method (Trinder)	mg/dL
Creatinine	Basques-Lustosa method	mg/dL
AP	Modified Roy’s method	U/L
Iron	Godwin method	µg/dL
Phosphorus	Phosphomolybdate method	mmol/L
Glucose	PAP enzymatic method	mg/dL
GGT	Modified Szaz method	U/L
Magnesium	Sulphonated carbon method	mg/dL
Potassium	Electrolyte analyser *	mmol/L
Total protein	Biuret method	g/dL
Sodium	Electrolyte analyser *	mmol/L
Triglycerides	Enzymatic method (Trinder)	mg/dL
Urea	Urease method	mg/dL

* Roche 9180 Electrolyte Analyser, Roche, São Paulo, BRA. ALT: alanine aminotransferase, AP: alkaline phosphatase, AST: aspartate aminotransferase, CK: creatinine kinase, g/dL: gram/deciliter, GGT: γ-glutamil transferase, mEq/L: miliequivalent/liter, mg/dL: milligram/deciliter, mmol/L: milimolar/liter, U/L: unity/liter, UV: ultraviolet.

**Table A2:** Maximum, mean, standard deviation, median and minimum for serum concentrations of metabolites of weaning beef calves at Brazilian savannah.

		Metabolites							
Breed		Creat (mg/dL)	Urea (mg/dL)	Chol (mg/dL)	Triglyc (mg/dL)	Gluc (mg/dL)	TB (mg/dL)	DB (mg/dL)	IB (mg/dL)
NE	Min	1.25	8	105	17	76	0.1	0.04	0.05
	Mean	1.53 *^,a^	16.61 ^b^	164.32 ^a^	28.55 ^a^	161.03 ^a^	0.21 ^b^	0.08 d	0.13 ^a^
	SD	0.13	4.82	30.32	8.66	53.40	0.09	0.03	0.07
	Median	1.55	15	168	27	154	0.17	0.08	0.1
	Max	1.83	26	231	46	304	0.44	0.16	0.32
SE	Min	0.97	14	38	11	78	0.18	0.16	0.01
	Mean	1.26 ^c^	21.73 ^a^	83.87 ^d^	18.90 ^b,c^	101.20 ^c^	0.32 ^a^	0.26 ^a^	0.06 ^c^
	SD	0.16	5.97	21.51	6.06	15.92	0.10	0.06	0.05
	Median	1.24	22	82.5	17	98.5	0.29	0.25	0.04
	Max	1.63	38	124	41	155	0.58	0.41	0.21
NE×AN	Min	1.02	6	78	9	76	0.12	0.09	0.01
	Mean	1.24 ^c^	15.94 ^b^	134.35 ^b^	21.13 ^b^	95.34 ^c^	0.26 ^a,b^	0.18 ^b,c^	0.08 ^b,c^
	SD	0.15	5.72	25.70	8.17	13.34	0.09	0.05	0.05
	Median	1.23	16	139	20	93	0.26	0.17	0.08
	Max	1.63	29	183	42	127	0.54	0.26	0.28
NE×SE	Min	0.84	8	66	8	75	0.1	0.08	0
	Mean	1.40 ^b^	21.35 ^a^	124.20 ^b,c^	15.80 ^c^	117.06 ^b^	0.25 ^a,b^	0.20 ^b^	0.06 ^c^
	SD	0.23	5.93	27.60	9.72	28.27	0.13	0.07	0.06
	Median	1.42	22.5	121.5	13.5	111	0.22	0.185	0.04
	Max	1.96	34	180	48	202	0.62	0.36	0.26
Tricross	Min	1.07	5	81	12	63	0.08	0.05	0
	Mean	1.53 ^a^	17.84	117.40 ^c^	22.97 ^b^	91.40 ^c^	0.26 ^a,b^	0.15 ^c^	0.11 ^a,b^
	SD	0.26	±5.41 ^b^	22.45	8.15	14.33	0.15	0.07	0.09
	Median	1.56	18	117	20	92	0.22	0.14	0.1
	Max	2.1	28	161	51	125	0.78	0.36	0.42

* Mean values followed by different letters in the columns differ by the Kruskall-Wallis test with *p* adjusted by the Benjamini-Hochberg method at 5% (*p* ≤ 0.05). Creat: creatinine, Chol: cholesterol, DB: direct bilirubin, Gluc: glucose, IB: indirect bilirubin, Max: maximum value, Median: median value, Min: minimum value, NE: Nellore breed, NE×AN: bicrossed by Nellore and Aberdeen Angus, NE×SE: bicrossed by Nellore and Senepol, SE: Senepol breed, SD: standard deviation, TB: total bilirubin, Tricross: triple-cross 50% Senepol, 25% Nellore, 25% Aberdeen Angus, Triglyc: triglycerides.

**Table A3:** Maximum, mean, standard deviation, median and minimum for serum electrolytes concentration of weaning beef calves at Brazilian savannah.

		Electrolytes							
Breed		Ca (mg/dL)	Ca i (mmol/L)	Cl^−^ (mmol/L)	Fe (µg/dL)	K (mmol/L)	Mg (mg/dL)	Na (mmol/L)	P (mg/dL)
NE	Min	8.2	3.16	97.96	76	3.32	2.4	134.73	6
	Mean	9.95 *^,a^	4.52 ^a^	102.66 ^a^	111.71 ^c^	4.06 ^a^	2.8 ^a^	142.40 ^a^	7.06 ^a^
	SD	0.6	0.3	1.91	24.18	0.52	0.2	2.72	0.91
	Median	10.1	4.59	102.6	109	3.89	2.8	143.02	7
	Max	11	4.96	107.45	173	5.42	3.2	150.48	10
SE	Min	9.2	3.85	97.21	83	3.39	1.6	136.53	4.9
	Mean	9.77 ^a^	4.60 ^a^	99.25 ^a^	156.33 ^a^	4.15 ^a^	1.97 d	139.26 ^b^	6.29 ^b^
	SD	0.36	0.28	1.27	28.22	0.51	0.17	1.77	0.66
	Median	9.7	4.67	99.23	159	3.95	2	138.96	6.2
	Max	10.5	5.17	102.6	214	5.98	2.3	143.02	7.8
NE×AN	Min	9.1	3.19	96.03	109	3.07	1.6	109.38	4.9
	Mean	9.86 ^a^	4.49 ^a^	102.18 ^a^	139.93 ^b^	3.78 ^b^	2.10 ^c^	138.47 ^b^	6.03 ^b^
	SD	0.34	0.38	10.16	28.48	0.23	0.20	6.11	0.70
	Median	9.9	4.6	100.76	130	3.79	2.1	139.77	6
	Max	10.6	5.03	155.98	207	4.3	2.5	144.36	7.7
NE×SE	Min	8.9	3.62	97.25	94.4	3.54	1.9	137.98	5
	Mean	9.76 ^a^	4.47 ^a^	101.70 ^a^	160.08 ^a^	4.07 ^a^	2.40 ^b^	142.10 ^a^	6.09 ^b^
	SD	0.41	0.30	1.61	27.43	0.45	0.40	1.96	0.64
	Median	9.8	4.55	101.27	160.2	3.93	2.4	141.89	6.05
	Max	10.7	4.96	104.39	258.2	5.35	4.37	146.55	7.3
Tricross	Min	8.8	3.54	99.24	59	3.46	2.1	132.45	6
	Mean	9.83 ^a^	4.38 ^a^	102.42 ^a^	115.10 ^c^	3.95 ^a,b^	2.44 ^b^	140.08 ^b^	7.06 ^a^
	SD	0.41	0.31	1.74	29.08	0.23	0.17	2.93	0.81
	Median	9.8	4.49	102.82	113	3.93	2.4	140.98	7
	Max	10.5	4.85	104.66	188	4.46	2.8	144.05	9

* Mean values followed by different letters in the columns differ by the Kruskall-Wallis test with *p* adjusted by the Benjamini-Hochberg method at 5% (*p* ≤ 0.05). Ca: calcium, Ca i: ionized calcium, Cl^−^: Chloride, Fe: iron, K: potassium, Max: maximum value, Median: median value, Mg: magnesium, mg/dL: milligram/deciliter, Min: minimum value, mmol/L: milimolar/deciliter, Na: sodium, NE: Nellore breed, NE×AN: bicrossed by Nellore and Aberdeen Angus, NE×SE: bicrossed by Nellore and Senepol, P: phosphorus, SE: Senepol breed, SD: standard deviation, Tricross: triple-cross 50% Senepol, 25% Nellore, 25% Aberdeen Angus, µg/dL: microgram/deciliter.
